# Excess mortality and underlying causes of death during the COVID-19 pandemic in rural Bangladesh: insights from the Matlab health and demographic surveillance system

**DOI:** 10.1186/s12963-025-00447-0

**Published:** 2026-03-19

**Authors:** Sayed Saidul Alam, Nur E Jannat Amee, Srizan Chowdhury, Md Mehedi Hasan, Chodziwadziwa Whiteson Kabudula, Jean Juste Harrisson Bashingwa, Md. Sharoardy Sagar, Munirul Alam Bhuiyan, M. Zahirul Haq, Beth A. Tippett Barr, Stephen Tollman, Syed Manzoor Ahmed Hanifi

**Affiliations:** 1 Health Systems and Population Studies Division, International Centre for Diarrhoeal Disease Research, Bangladesh (icddr,b), Dhaka, Bangladesh; 2 SAMRC/Wits Rural Public Health and Health Transitions Research Unit (Agincourt), School of Public Health, Faculty of Health Sciences, University of the Witwatersrand, Johannesburg, South Africa; 3 Nyanja Health Research Institute, Salima, Malawi

**Keywords:** COVID-19, Mortality, Elderly, HDSS, Matlab, Bangladesh

## Abstract

**Background:**

Bangladesh, home to 165 million people, reported its first COVID-19 case in March 2020. This prompted a range of public health measures to control the epidemic. However, limited access to COVID-19 testing and incomplete or inaccurate death registration likely obscured the pandemic’s true impact. We use longitudinal data from the Matlab Health and Demographic Surveillance System (HDSS) in Bangladesh to assess excess mortality and underlying causes of death during the COVID-19 pandemic.

**Methods:**

We analysed mortality among 299,775 individuals residing within the Matlab HDSS catchment area between January 1, 2018 and December 31, 2021. Crude mortality rates were compared between the Pre-COVID-19 (2018–2019) and COVID-19 (2020–2021) periods. Adjusted sub-distribution hazard ratios (SHR) were estimated using the Fine and Gray competing risk model. Causes of death were determined using the WHO 2016 Verbal Autopsy questionnaire with supplementary COVID-19 module. We assessed changes in the distribution of causes of death and calculated cause-specific mortality rates by period and sex.

**Results:**

Crude mortality rate increased to from 7.4 deaths per 1000 person-years in 2018–2019 (pre–COVID-19 period) to 8.5 deaths per 1000 person-years during the COVID-19 period (2020–2021). Among individuals aged 60 years and above, the COVID-19-related mortality rate was 3.5 deaths per 1000 person-years during the COVID-19 period. Overall mortality rate increased from 44.1 (95% CI: 42.4–45.9) deaths to 50.9 (95% CI: 49.1–52.7) deaths per 1000 person-years, corresponding to an adjusted SHR of 1.19 (95% CI: 1.12–1.25). Compared with the Pre-COVID-19 period, mortality attributable to non-communicable diseases (NCDs) increased by 11% (mortality rate ratio (MRR): 1.11; 95% CI: 1.04–1.18), while mortality from respiratory diseases increased by 82% (MRR: 1.82; 95% CI: 1.24–2.73) during the COVID-19 period.

**Conclusion:**

During the COVID-19 period, mortality increased in rural Bangladesh, with the sharpest increase observed among older adults with noncommunicable and respiratory diseases. Future pandemic preparedness efforts should prioritise these high-risk subgroups to reduce adverse health outcomes and mortality.

**Supplementary Information:**

The online version contains supplementary material available at 10.1186/s12963-025-00447-0.

## Introduction

The COVID-19 pandemic caused a significant increase in global mortality [1]. Several studies have reported excess mortality in countries across the world, providing insights into the pandemic’s global impact [2]. The number of global excess deaths was estimated to be around 14.83 million, which is 2.74 times higher than the reported 5.42 million COVID-19 deaths during the same period [3–5].

Although many Asian countries reported official COVID-19 death counts [6], it is increasingly evident that the figures do not provide a complete picture of the pandemic’s true impact on mortality. Limited testing and weak reporting systems in low and middle-income countries (LMICs), such as Bangladesh [7], likely led to significant underestimation of the magnitude of COVID-19-related mortality.

Healthcare systems, being at the forefront of the crisis, experienced significant service delivery interruptions [8] that resulted in restricted availability of routine and vital medical treatments for many persons. Healthcare systems, initially overwhelmed by the surge in COVID-19 cases, struggled to maintain their regular services [9–11]. Routine and critical medical treatments became less accessible, causing delayed diagnoses and treatments for various health conditions [11, 12]. The impact of the pandemic significantly varied between urban and rural environments. In rural areas, limited healthcare resources exacerbated challenges in testing, treatment, and disease control [13].

Health and demographic surveillance systems (HDSSs) are uniquely positioned to address evidence gaps in the identification of complex patterns of mortality associated with pandemics. This is particularly important in areas where there is insufficient recording of vital statistics data and where reports of deaths occurring within healthcare facilities may under-report mortality [14], especially in contexts where deaths which occur at home are not registered or counted [13]. Since HDSSs monitor vital events in populations in defined geographic areas for extended periods of time, in the past, they have provided estimates of vital events for settings in several LMICs where Civil Registration and Vital Statistics (CRVS) systems are weak or incomplete [13].

This study uses longitudinal data from the Matlab HDSS, which has been running since 1966 by the International Centre for Diarrhoeal Disease Research, Bangladesh (icddr,b), to assess the mortality impact of COVID-19 and identify the causes contributing to excess mortality. Being one of the world’s longest-running HDSS, the Matlab HDSS has historically generated key information on demographic and mortality events in Bangladesh. Although prior estimates quantified excess mortality in 2020 compared with 2018–2019 [13], the impact of COVID-19 over the period 2020–2021 and the causes of excess mortality are still unexplored. This study, therefore, aims to gain an extensive understanding of the particular effects of COVID-19 on mortality and the underlying causes contributing to excess deaths.

## Materials and methods

### Study setting

This study utilised data from the Matlab HDSS operated by icddr,b in a rural area in Bangladesh [15]. Established in 1966, the Matlab HDSS, currently collects longitudinal data on health, demographic, and socio-economic characteristics within a defined population of 246,893 individuals residing in 59,890 households spread across 142 rural villages approximately 55 km southeast of Dhaka. The data is collected by community health research workers (CHRWs) using tablets that are connected to mobile internet through the network of local mobile operators. Each household is visited every three months, during which CHRWs record health and demographic events of household members. The collected data is immediately uploaded to a central icddr,b database server [13].

### Study population

**Fig. 1:**
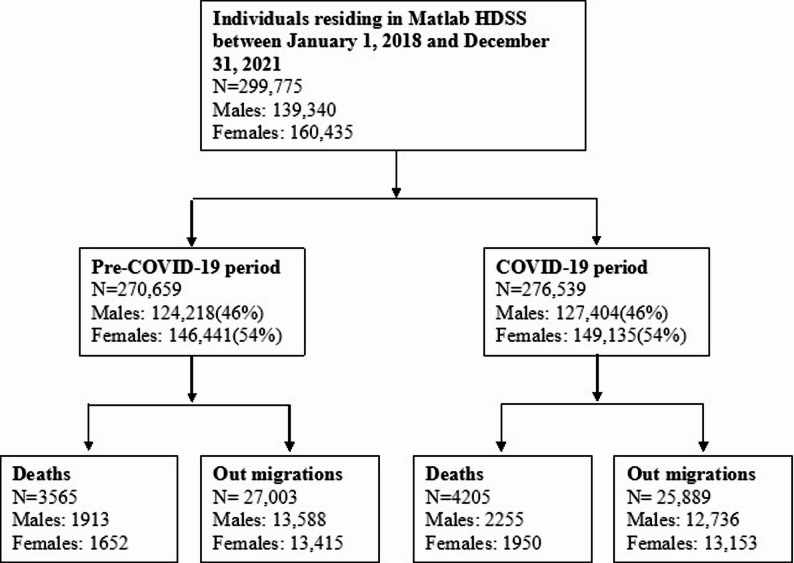
Flowchart of study population

We followed 299,775 individuals residing in the Matlab HDSS between January 1, 2018 and December 31, 2021. During the Pre-COVID-19 period (2018–2019), 27,003 individuals migrated out and 3,565 deaths were recorded among 270,659 individuals. In the COVID-19 period (2020–2021), 25,889 individuals migrated out and 4,205 deaths were recorded among 276,539 individuals. A summary of the study population is presented in Fig. 1.

### Variables and measurement

For each individual residing in the Matlab HDSS between January 1, 2018 and December 31, 2021, we retrieved data on date of birth, sex, marital status, level of education, date of entry into the HDSS population and date of exit (including death or outmigration) from the Matlab HDSS database. Data on durable and traditional household assets, including radio/DVD/VCD player, television, mobile/land telephone, refrigerator, almirah/showcase, electric fan, fishing boat, water pump, sewing machine, computer/laptop, IPS/generator, auto-bike/tempo/CNG-auto-rickshaw, rickshaw/van, bicycle, motorcycle, homestead land, agricultural land, cows/buffaloes, goats/sheep and poultry (chickens/ducks) was used to create a composite score for household socioeconomic status. The score was created using principal component analysis and wealth quintiles were obtained by dividing the ranked scores into five equal categories, each representing 20% of households.

For each recorded death, the data also included information on causes of death. The information was collected using the WHO 2016 Verbal Autopsy (VA) instrument [16], with an additional COVID-19 section added in 2020. The VA instrument was implemented in a web-based software application developed for the Matlab HDSS. Trained field research supervisors (FRS) conducted the VA interviews with close relatives of the deceased at least seven days after death. Medical personnel used the VA data to assign causes of death following ICD-10 guidelines [17].

## Epidemiological and Statistical Methods

We defined 2018–2019 as the Pre-COVID-19 and 2020–2021 as the COVID-19 period. We calculated crude mortality rates (CMRs) by age and sex as COVID-19 mortality is known to vary by these factors [18, 19]. Age-specific and sex-specific person-years were used as denominators in computing the rates which were then compared across the two COVID-19 periods.

In the context of this study, individuals could reach one of three possible end-points: death, out-migration and end of follow-up. When estimating mortality in a survival analysis framework, death would be the event of interest. A standard Cox proportional hazards model would treat death as the only event of interest and both out-migration and end of follow-up as censoring. This would require out-migration to be ‘non-informative’ and not influence the statistical distribution of mortality. However, in the context of this study, out-migration may be selective on unobserved characteristics related to the health of the participants. During the COVID-19 era, prolonged lockdowns likely restricted population movements in and out of the study area. Either, the majority of the healthier individuals could no longer out-migrate or the very sick would out-migrate to seek treatment. These contrasting migration patterns could induce bias in the estimation of mortality before and during the COVID-19 pandemic. This can be addressed by treating out-migration as a competing event other than censoring.

Fine and Gray’s [20] competing risk regression model accounts for the influence of competing events by modelling the effects of covariates on the Cumulative Incidence Function (CIF), which represents the probability of an event occurring within a specified time. The model produces sub-distribution hazards and sub-distribution hazard ratios (SHRs) for covariates, analogous to hazard ratios (HRs) in a Cox model. Austin and Fine [21] published further details on the practical application of the Fine and Gray competing risk regression, including interpretation of model outputs.

To mitigate any potential selection bias due to in-migration, we included a ‘migration status’ variable in the competing risk regression model. This variable, defined by migratory behavior of individuals over the study period, comprised four categories: (a) non-migrants (reference category) - individuals continuously present in the HDSS area from January 1, 2018 and followed until death, a singular out-migration, or the end of the study period (December 31, 2021); (b) new in-migrants - individuals who entered the HDSS area for the first time during the study period, without prior residency or multiple in-migrations; (c) return in-migrants - individuals who re-entered the HDSS area during the study period, having lived there previously, but without multiple in-migrations; and (d) circular migrants- individuals with two or more in- or out-migrations during the study period, indicating a circular migratory pattern.

We included sex, level of education, marital status, and SES as potential confounders in the models for adjusted estimates. For the cause of death analysis, we calculated the percentage distribution of cause-specific mortality rates by age, sex and period. We compared the cause-specific mortality rates between the pre-COVID-19 and COVID-19 periods by sex and age. Analyses were conducted using STATA 17 [22] and a figure with a map was produced with ArcGIS 10.

## Results

### Mortality

**Fig. 2:**
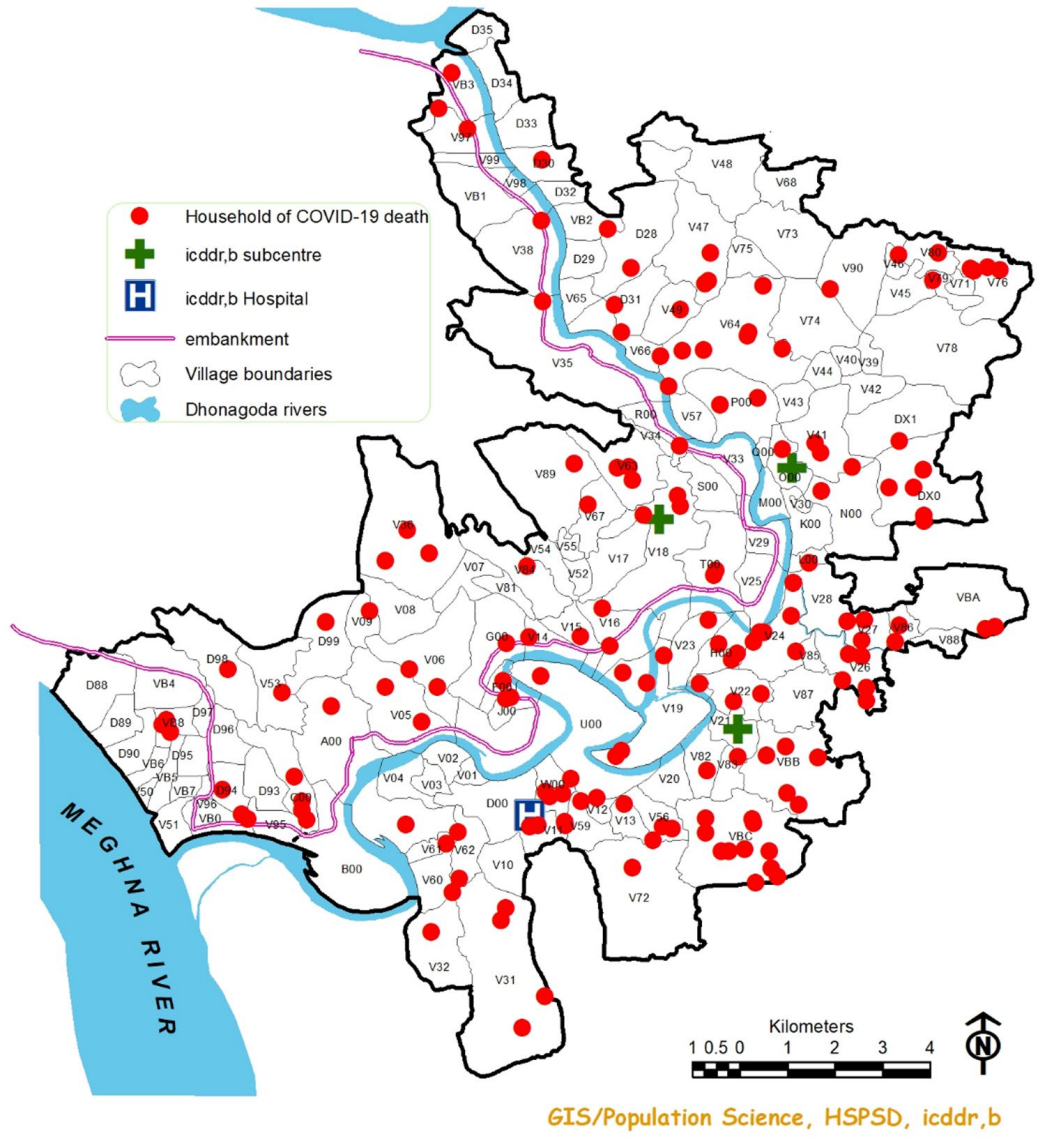
Distribution of households with COVID-19 death, Matlab HDSS area

Between 2018 and 2021, a total of 7,770 deaths were recorded in the Matlab HDSS: 3,565 occurred before the COVID-19 pandemic (2018–2019) and 4,205 during the COVID-19 pandemic (2020–2021). Of the latter, 287 deaths were directly attributed to COVID-19, distributed across all 142 villages (Fig. 2).

Figure 2 shows the distribution of COVID-19 deaths in the Matlab HDSS area during the COVID-19 pandemic. The deaths occurred across 59,890 households distributed across all 142 villages of the study area.

COVID-19 mortality rates were 18 deaths per 100,000 person-years among individuals younger than 60 years and as high as 350 deaths per 100,000 person-years among those aged 60 years and older, corresponding to an MRR of 19.26 (95% CI: 14.80–25.28).

Between 2020 and 2021, there were 416 excess deaths, of which half (*n* = 208) were attributed to COVID-19. In addition, non-communicable diseases (NCDs) and respiratory diseases also contributed to the rise in mortality among older adults.

Table 1 presents crude mortality rates (CMR) by age, sex, and period (pre-COVID-19 vs. COVID-19). The overall crude death rate increased from 7.4 to 8.5 per 1000 person-years between the two periods.

**Table 1:** Crude mortality rate (CMR) per 1, 000 person-years (pyrs) according to age and period

Age group (in years)	Male		Female		Total	
	Pre-COVID-19	COVID-19	Pre-COVID-19	COVID-19	Pre-COVID-19	COVID-19
< 10	4.09* [3.57, 4.69]** (50590)***	3.84 [3.34, 4.41] (51598)	2.90 [2.46, 3.41] (49998)	2.73 [2.31, 3.22] (50630)	3.50 [3.15, 3.88] (101588)	3.29 [2.95, 3.66] (102228)
10–19	0.72 [0.51, 1.02] (44256)	0.62 [0.43, 0.90] (45135)	0.51 [0.35, 0.76] (48751)	0.81 [0.59, 1.10] (48334)	0.61 [0.47, 0.79] (93007)	0.72 [0.56, 0.91] (93469)
20–29	1.13 [0.77, 1.68] (22070)	0.91 [0.60, 1.38] (24217)	0.68 [0.46, 0.99] (39902)	0.67 [0.46, 0.98] (40363)	0.84 [0.64, 1.10] (61972)	0.76 [0.57, 1.00] (64580)
30–39	1.41 [1.01, 1.96] (24894)	1.48 [1.08, 2.02] (26434)	1.04 [0.76, 1.43] (36505)	1.40 [1.07, 1.84] (37764)	1.19 [0.95, 1.50] (61399)	1.43 [1.17, 1.76] (64198)
40–49	4.42 [3.65, 5.36] (23738)	3.81 [3.12, 4.66] (24908)	2.18 [1.71, 2.77] (30285)	2.19 [1.72, 2.78] (30632)	3.17 [2.72, 3.68] (54023)	2.92 [2.50, 3.40] (55540)
50–59	10.65 [9.45, 12.01] (25065)	10.21 [9.03, 11.54] (24986)	5.91 [5.08, 6.86] (28955)	7.27 [6.37, 8.30] (29992)	8.11 [7.38, 8.90] (54020)	8.60 [7.86, 9.41] (54978)
*≥* 60	47.61 [45.03, 50.33] (26087)	56.64 [53.94, 59.47] (28567)	40.93 [38.66, 43.34] (28827)	45.57 [43.25, 48.01] (30897)	44.10 [42.38, 45.90] (54914)	50.89 [49.11, 52.73] (59464)
Overall	8.82 [8.44, 9.23] (216700)	9.98 [9.58, 10.41] (225845)	6.28 [5.98, 6.59] (263223)	7.26 [6.94, 7.59] (268612)	7.43 [7.19, 7.68] (479923)	8.50 [8.25, 8.77] (494457)

* Crude mortality rate

** 95% confidence intervals

*** Person-years

**Fig. 3:**
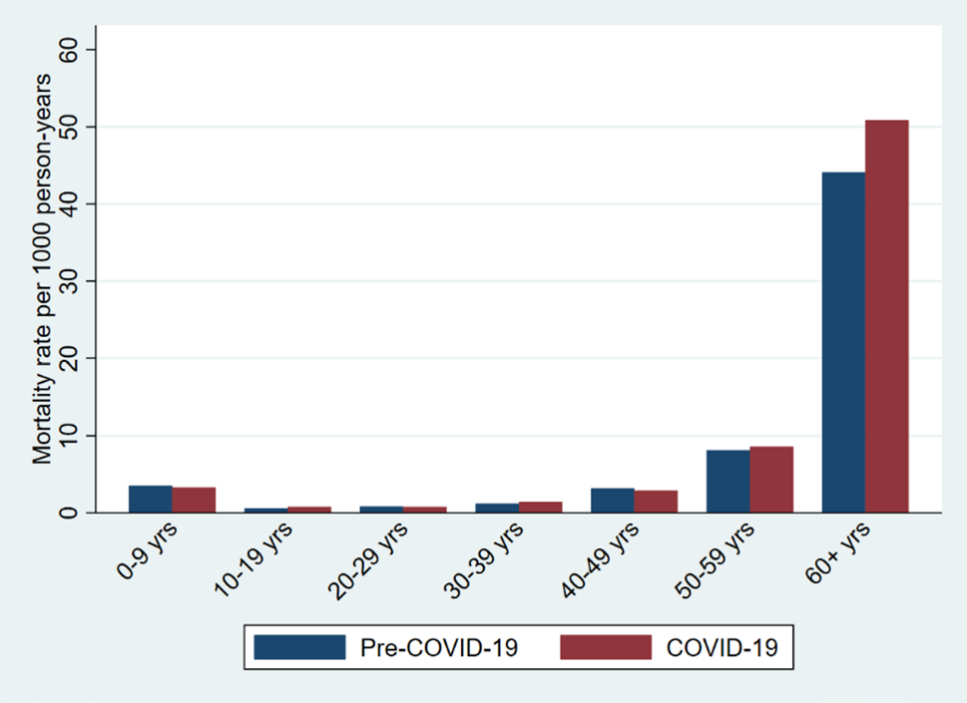
Mortality rate between Pre-COVID-19 and COVID-19 by age

**Fig. 4:**
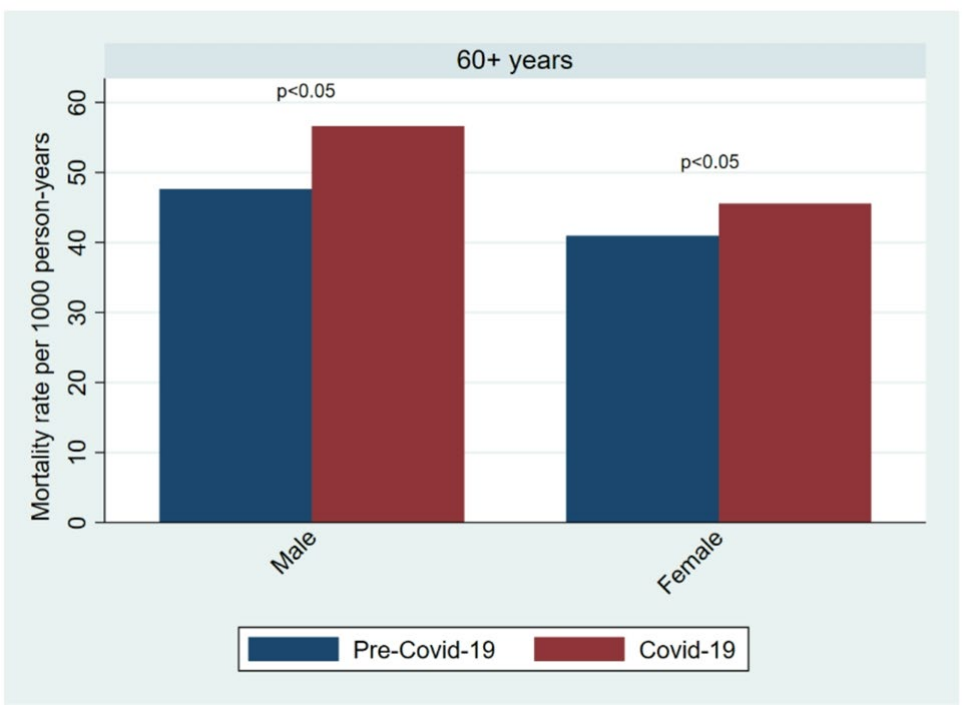
Mortality rate among individuals aged 60 years and over by gender and period

The mortality rate among individuals younger than 60 years remained stable at 2.7 deaths per 1000 person-years across both periods. In contrast, the CMR among those aged 60 years and older increased from 44.1 deaths per 1000 person-years in the pre-COVID-19 period to 50.9 deaths per 1000 person-years during COVID-19 (Table 1; Fig. 3). Excess deaths were observed among both males and females. Among males, mortality increased from 47.6 to 56.6 deaths per 1000 person-years, corresponding to an adjusted SHR of 1.24 (95% CI: 1.15–1.34) whereas among females, mortality increased from 40.9 to 45.6 deaths per 1000 person-years, resulting in an adjusted SHR of 1.13 (95% CI: 1.05–1.23) (Fig. 4; Table 2). The adjusted model showed that individuals who in-migrated more frequently during the study period had lower mortality rate compared with non-migrants. Specifically, the SHR was 0.91 (95% CI: 0.72–1.16) for new in-migrants, 0.80 (95% CI: 0.61–1.04) for return in-migrants and 0.20 (95% CI: 0.13–0.30) for circular migrants. Mortality among circular-migrants was one-fifth of non-migrants (p-value < 0.001), but the difference in excess mortality between men and women across the two periods was not statistically significant (*P* = 0.06).

**Table 2:** Crude and adjusted sub-distribution hazard ratio (SHR) for the COVID-19 era and individuals aged 60 years and above by sex

	Male			Female			Overall		
	MR (Deaths/person-yrs)	Crude SHR (95%CI)	Adj. SHR*	MR (Deaths/person-yrs)	Crude SHR (95%CI)	Adj. SHR*	MR (Deaths/person-yrs)	Crude SHR (95%CI)	Adj. SHR*
Pre COVID-19	47.61 (1242/ 26087)	(Ref)	(Ref)	40.93 (1180/ 28827)	(Ref)	(Ref)	44.11 (2422/54915)	(Ref)	(Ref)
COVID-19	56.64 (1618/ 28568)	1.24 (1.15, 1.34)	1.24 (1.15, 1.34)	45.57 (1408/ 30897)	1.14 (1.05, 1.23)	1.13 (1.05, 1.23)	50.89 (3026/59465)	1.19 (1.13, 1.26)	1.19 (1.12, 1.25)

*Adjusted for area, marital status, education, wealth, and migration status

**Fig. 5:**
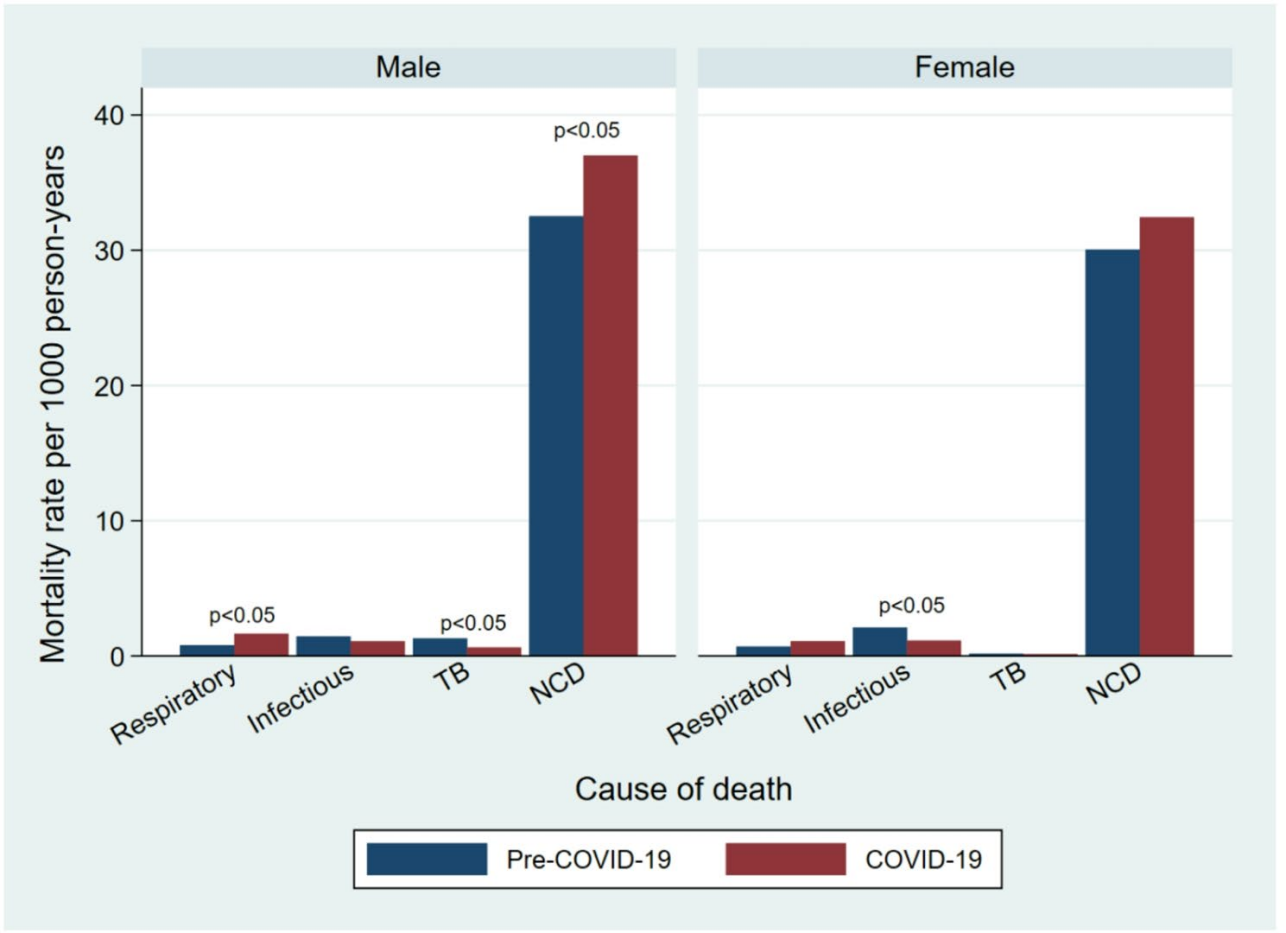
Cause-specific mortality rates among individuals aged 60 years and over by sex and period

### Causes of death

Table 3 presents the causes of death among individuals aged 60 years and older before and during the COVID-19 pandemic. Overall patterns were similar across the two time periods, but notable increases were observed in mortality from NCDs and respiratory diseases. Between the pre-COVID-19 and COVID-19 periods, the death rate per 1000 person-years attributed to NCDs increased from 31.2 to 34.6, corresponding to an MRR of 1.11 (95% CI: 1.04–1.18) (Fig. 5 & Additional file 1). Mortality from respiratory diseases increased from 0.8 to 1.4 deaths per 1000 person-years, resulting in an MRR of 1.82 (95% CI: 1.24–2.73) (Fig. 5 & Additional file 1). By contrast, mortality rate from infectious diseases decreased from 1.8 to 1.1 deaths per 1000 person-years (MRR: 0.62; 95% CI: 0.44–0.85) and from pulmonary TB decreased from 0.7 to 0.4 deaths per 1000 person-years (MRR: 0.52; 95% CI: 0.29–0.90) (Fig. 5 & Additional file 1). Among individuals younger than 60 years, no significant difference in cause-specific mortality were observed between the two periods (Fig. 3).

**Table 3:** Causes of death before COVID-19 and during COVID-19 among individuals aged 60 years and above

Broad Disease Group (ICD-10)	Pre-COVID-19 (2018–2019)			COVID-19 (2020–2021)		
	Male [*n*(%)]	Female [*n*(%)]	Both sex [*n*(%)]	Male [*n*(%)]	Female [*n*(%)]	Both sex [*n*(%)]
Respiratory diseases (J00-J42, J48-J99)	21(1.7)	20(1.7)	41(1.7)	47(3.1)	34(2.6)	81(2.9)
Infectious diseases (A00-A14, A20-A99, B00-B99)	38(3.1)	61(5.2)	99(4.1)	31(2.1)	35(2.6)	66(2.3)
Pulmonary TB. (A15-A19, B90)	34(2.7)	5(0.4)	39(1.6)	18(1.2)	4(0.3)	22(0.8)
Neoplasm (C00-C99)	154(12.4)	66(5.6)	220(9.1)	158(10.6)	68(5.1)	226(8.0)
NCD (I00-I99, E10-E14, J43-J47)	848(68.2)	866(73.4)	1714(70.7)	1057(70.7)	1002(75.8)	2059(73.1)
Maternal and Neonatal (O00-O99, P00-P99)	0(0.0)	0(0.0)	0(0.0)	0(0.0)	0(0.0)	0(0.0)
External (V00-V99, W00-W99, X00-X99, Y00-Y99)	37(3.0)	48(4.1)	85(3.5)	43(2.9)	46(3.5)	89(3.2)
Indeterminate (R00-R99)	44(3.5)	67(5.7)	111(4.6)	63(4.2)	101(7.6)	164(5.8)
Miscellaneous	67(5.4)	47(4.0)	114(4.7)	79(5.3)	32(2.4)	111(3.9)
Total	1243(100)	1180(100)	2423(100)	1496(100)	1322(100)	2818(100)

## Discussion

The elderly population, aged 60 years and above, experienced the high risk of dying during the COVID-19 pandemic. The results of our study are consistent with global estimates that indicate a higher mortality rate among those aged 60 years and older as a result of the COVID-19 pandemic [23]. While global data indicates that individuals within this age group accounted for more than 80% of total COVID-19-related deaths, our analysis produced a slightly lower estimate of 72% [23]. Excess mortality, which is a robust measure of the pandemic’s overall impact, indicates that only 50% of these deaths were directly attributable to COVID-19.

Our data also revealed an increase in NCD related mortality over the years 2020 and 2021. This rise can potentially be attributed to the complex interaction between COVID-19, pre-existing NCDs, healthcare system constraints during the pandemic [24]. People living with NCDs experienced greater difficulties during the epidemic, including the negative impacts of quarantine, poor diets, and reduced physical activity [25–27]. The redirection of healthcare resources and personnel to address COVID-19 led to considerable disruptions in access to essential medications and treatments for NCD patients, particularly in areas with prolonged lockdowns [26, 27]. As a result, many experienced declines in both health status and quality of life. In a country like Bangladesh, where healthcare services were already under strain, the impacts of these interruptions became even more pronounced and may have contributed to higher mortality among individuals living with NCDs.

Our analysis also revealed an increase in mortality from respiratory disorders throughout the COVID-19 period. This finding is consistent with research conducted in Pakistan, which reported a heightened risk of mortality among individuals co-infected with tuberculosis and COVID-19 [28]. The observed age-related trend in mortality is also consistent with the global data on COVID-19 excess mortality [29–32], emphasizing the heightened risk of mortality associated with older age.

The interaction between COVID-19 and pre-existing health issues in Bangladesh highlights the need for implementing a comprehensive health approach. Protecting vulnerable populations, particularly the elderly, must be a priority to strengthen epidemiological preparedness for future pandemics.

The findings of this study underscore the need for targeted interventions to protect the vulnerable elderly population during health crises. It is crucial to prioritize the development and implementation of measures that aim to mitigate the impact of the pandemic on the elderly population’s health outcomes. These measures may include improved access to healthcare services, increased surveillance and monitoring, and targeted vaccination campaigns.

A key strength of this study was the use of population-based health and demographic surveillance data, which enabled precise population-based estimates of excess mortality in the Matlab HDSS area. However, a limitation is that these estimates are based on only one rural area and may not fully represent rural Bangladesh, especially since the Matlab HDSS area has been documented to have better health outcomes than many other rural areas of the country [13].

## Conclusions

During the COVID-19 outbreak, a noticeable excess in deaths was observed in the study area, especially among older people. In addition to COVID-19 itself, NCDs and respiratory diseases were the major contributors to this excess mortality. While the WHO has highlighted that older people with pre-existing health conditions are more likely to get sick, our findings suggest a potential deficiency in implementing protective measures tailored to Bangladesh’s context. For future outbreaks, health policy frameworks must prioritise the safety of older people, especially those with underlying health problems. This will require greater investments in strengthening Bangladesh’s health infrastructure and ensuring that healthcare services are accessible to all population sub-groups.

## Supplementary Information

Supplementary Material 1.

## Data Availability

Data used in this paper is made available on https://data.agincourt.co.za/index.php/catalog/334.

## Abbreviations

HDSS: Health and Demographic Surveillance System
icddr,b: International Centre for Diarrhoeal Disease Research, Bangladesh
CRVS: Civil Registration and Vital Statistics
VA: Verbal Autopsy
ICD: International Classification of Diseases
NCD: Noncommunicable diseases
LMIC: Low- and Middle-Income Country
MR: Mortality rate
MRR: Mortality rate ratio
CMR: Crude mortality rate
SES: Socioeconomic status
CHRW: Community Health Research Worker
FRS: Field Research Supervisor
DVD: Digital Video Disc
VCD: Video Compact Disc
CNG: Compressed Natural Gas
IPS: Instant Power Supply
