# Supplementary material for: Excess mortality and underlying causes of death during the COVID-19 pandemic in rural Bangladesh: insights from the Matlab health and demographic surveillance system
Source: Popul Health Metr. 2026 Mar 19;23(Suppl 2):77. doi: 10.1186/s12963-025-00447-0 (PMC13003680; doi:10.1186/s12963-025-00447-0)
Supplement: Supplementary file 1 — Supplementary Material 1. [file 12963_2025_447_MOESM1_ESM.docx]

**Additional file 1.** Comparison of cause-specific mortality rates for all the categories of causes by sex and period among individuals aged 60 years and above

|  | **Male** | | |  | **Female** | | |  | **Both Sex** | | |
| --- | --- | --- | --- | --- | --- | --- | --- | --- | --- | --- | --- |
| **Broad Disease Group** | **Pre- COVID-19** | **COVID-19** | **MRR (95% CI)** |  | **Pre- COVID-19** | **COVID-19** | **MRR (95% CI)** |  | **Pre- COVID-19** | **COVID-19** | **MRR (95% CI)** |
|  | **[n(%)]** | **[n(%)]** |  |  | **[n(%)]** | **[n(%)]** |  |  | **[n(%)]** | **[n(%)]** |  |
| Respiratory diseases | 21(1.7) | 47(3.1) | **2.04(1.20-3.60)** |  | 20(1.7) | 34(2.6) | 1.59(0.89-2.91) |  | 41(1.7) | 81(2.9) | **1.82(1.24-2.73)** |
| Infectious diseases | 38(3.1) | 31(2.1) | 0.74(0.45-1.23) |  | 61(5.2) | 35(2.6) | **0.54(0.34-0.82)** |  | 99(4.1) | 66(2.3) | **0.62(0.44-0.85)** |
| Pulmonary TB | 34(2.7) | 18(1.2) | **0.48(0.26-0.88)** |  | 5(0.4) | 4(0.3) | 0.75(0.15-3.47) |  | 39(1.6) | 22(0.8) | **0.52(0.29-0.90)** |
| Neoplasm | 154(12.4) | 158(10.6) | 0.94(0.75-1.18) |  | 66(5.6) | 68(5.1) | 0.96(0.67-1.37) |  | 220(9.1) | 226(8.0) | 0.95(0.78-1.15) |
| NCD | 848(68.2) | 1057(70.7) | **1.14(1.04-1.25)** |  | 866(73.4) | 1002(75.8) | 1.08(0.98-1.18) |  | 1714(70.7) | 2059(73.1) | **1.11(1.04-1.18)** |
| Maternal and Neonatal | 0(0.0) | 0(0.0) | - |  | 0(0.0) | 0(0.0) | - |  | 0(0.0) | 0(0.0) | - |
| External | 37(3.0) | 43(2.9) | 1.06(0.67-1.69) |  | 48(4.1) | 46(3.5) | 0.89(0.58-1.37) |  | 85(3.5) | 89(3.2) | 0.97(0.71-1.32) |
| Indeterminate | 44(3.5) | 63(4.2) | 1.31(0.88-1.97) |  | 67(5.7) | 101(7.6) | **1.41(1.02-1.95)** |  | 111(4.6) | 164(5.8) | **1.36(1.07-1.75)** |
| Miscellaneous | 67(5.4) | 79(5.3) | 1.08(0.77-1.51) |  | 47(4.0) | 32(2.4) | **0.64(0.39-1.02)** |  | 114(4.7) | 111(3.9) | 0.90(0.69-1.18) |
| Total: | 1243(100) | 1496(100) | **1.10(1.02-1.19)** |  | 1180(100) | 1322(100) | 1.05(0.97-1.13) |  | 2423(100) | 2818(100) | **1.07(1.02-1.13)** |

***^+^ p<0.05***
